# Evaluation of ex vivo effectiveness of commercial desensitizing dentifrices

**DOI:** 10.4317/jced.53040

**Published:** 2017-04-01

**Authors:** Hanny Mockdeci, Hudson Polonini, Isadora Martins, Ana-Paula Granato, Nádia Raposo, Maria-das Graças Chaves

**Affiliations:** 1 Faculty of Dentistry, Federal University of Juiz de Fora, Juiz de Fora, Brazil; 2 Faculty of Pharmacy, Federal University of Juiz de Fora, Juiz de Fora, Brazil

## Abstract

**Background:**

Dentin hypersensitivity is a short, severe pain with fast onset. Therapy aims to either prevent or decrease neural transmission or physically occlude the dentinal tubules. This study aimed to evaluate the effectiveness of commercial desensitizing dentifrice by means of an *ex vivo* method.

**Material and Methods:**

Samples (n=8 lower human premolars for each group) were randomly allocated into: G1- brushing with Colgate®Sensitive Pro-Relief; G2- brushing with Sensodyne®Rapid Relief; G3- brushing with Sensodyne®Repair & Protect; and G4- brushing with Colgate®Maximum Cavity Protection. The test bodies were submitted to simulated toothbrushing and dentifrices were analyzed regarding their hydrodynamic size, polydispersity index (PI) and zeta potential. Specimens were evaluated using: scanning electron microscopy (SEM); spectroscopy energy dispersive X-ray (EDS); and profilometry. A qualitative analysis of the photomicrographs and topographies was performed.

**Results:**

The dentifrices showed statistical similar physical and chemical characteristics. They also demonstrated obliteration of dentinal tubules when micrographs were observed. Regarding the chemical elements present in the dentin samples, there was a statistically significant difference between the control and experimental surfaces in the four groups.

**Conclusions:**

Joint data analysis shows that the desensitizing dentifrice showed better results with regards to the obliteration of dentinal tubules compared to positive and negative controls.

** Key words:**Dentin hypersensitivity, dentin desensitizing agents, toothpastes.

## Introduction

Improvements in the oral health status of the worldwide population are currently noteworthy, as well as the efficacy of dental treatments, which leads to lower rates of caries and periodontal diseases, thus contributing to the maintenance of the teeth in the oral cavity for longer (1). These factors, combined with the longer life expectancy of the world population, can indirectly lead to increased rate of dentin hypersensitivity (DH), since the higher the individual’s age and the number of teeth he has, the greater the chances of having DH (1,2). DH is described as a short, severe pain with fast onset, which may be either localized or generalized (3,4). It is a commonly misdiagnosed and improperly treated clinical condition. The prevalence rates of this condition vary from 3% to 73% of the adult population in Western Europe and in the United States of America, and worldwide incidence varies from 10% to 30% (5). There are a number of treatments for DH, which can be either pharmacological or non-pharmacological, either for home use or use by professionals. Namely, we can cite the following applications: high or low intensity laser (4), fluoride varnish (6), oxalate (6,7), composite resin (5) and glass ionomer (5), in addition to periodontal surgeries (5,8). However, the use of desensitizing dentifrices is the first choice for most patients (1,4,6,9). It is noteworthy that these are dose dependent, requiring routine applications (10). Another important fact is that dentin, when hypersensitive, features tubules with diameters of 0.83 µm, i.e., the dentifrices should ideally have particles with an average diameter such that they can penetrate the dentinal tubules, thus facilitating the release of chemicals which will bind to dentin (1,4,8). To the best of the authors’ knowledge, studies evaluating the size and stability of the particles with regards to commercial desensitizing dentifrices have not yet been conducted. Given the foregoing, the present study aimed to: (i) physically and -chemically assess four brands of commercial desensitizing dentifrice, in terms of their average particle size and surface charge; and (ii) determine their *ex vivo* effectiveness with regards to the obliteration process of the dentinal tubules.

## Material and Methods

-Physical and chemical analysis

Dynamic light scattering and electrophoretic mobility were used, respectively, to determine both the average hydrodynamic size and the zeta potential of particles using a Zetatrac equipment (Microtac, United States). Measurements were carried out at 25°C, after appropriate dilution (final concentration = 250 mg L-1) of samples in ultrapure water. The polydispersity index (PI) was calculated from the results regarding particle hydrodynamic diameter, in order to provide information about their size homogeneity. The results were obtained by calculating the average of the three readings.

-Determination of *ex vivo* effectiveness

•Preparation of dentin samples

**Figure 1:**
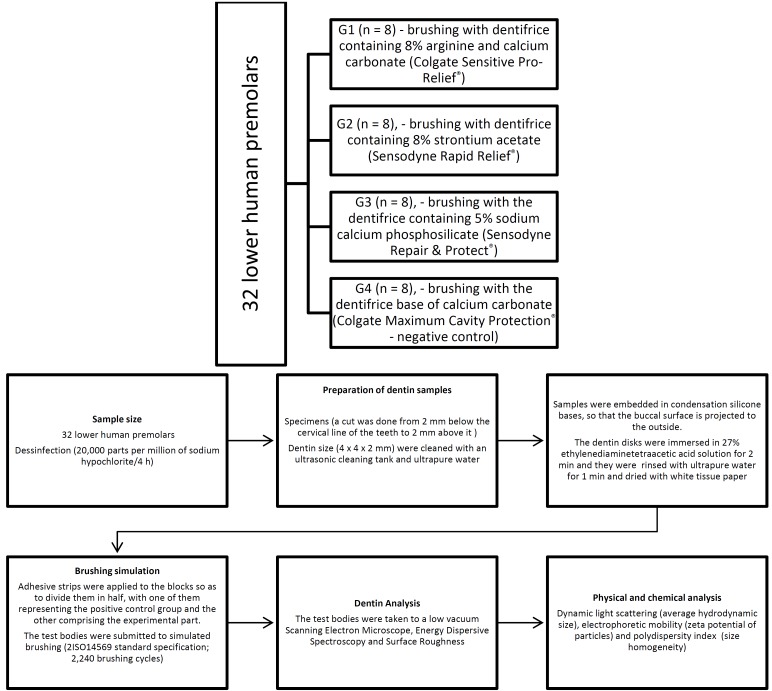
*Ex vivo* effectiveness study design.

In order to develop this study, 32 lower healthy human premolars were used (Fig. 1). They were extracted based on on clinical indication and acquired from the Tooth Bank of the Federal University of Juiz de Fora School of Dentistry, on the grounds that, as reported by various authors, these teeth are the most commonly affected by dentin hypersensitivity (6,11). All testing protocols were previously approved by the Ethics Committee of the Federal University of Juiz de Fora under opinion no. 810,805. Teeth were disinfected with 20,000 parts per million (ppm) of sodium hypochlorite for 24 hours at room temperature (12). Then a cut was done from 2 mm below the cervical line of the teeth to 2 mm above it with the aid of a metallographic cutter (Isomet® 1000 Precision Saw, Buehler, United States). The buccal surface of the specimen was abraded using a belt sander (Politriz PL02, Te-clago, Brazil), with decreasing grit sandpaper (1200, 2400 and 4000) under digital pressure to the exposure of the dentin surface (13). Then, the specimens were sectioned so that they reached the dimensions 4 x 4 x 2 mm (4,14). The specimens were cleaned with an ultrasonic cleaning tank (USC1400, Unique, Brazil) and ultrapure water (Direct-Q®, Millipore, France) for three cycles of 10 minutes each, in order to remove impurities, followed by washing by ultrapure water three more times (2). Subsequently, samples were embedded in condensation silicone (Speedex- Denso e catalizador, Coltene, Brazil) bases, so that the buccal surface is projected to the outside. In order to remove the smear layer and clean the surface, as well as simulate a hypersensitive dentin, the dentin disks were immersed in 27% ethylenediaminetetraacetic acid solution for 2 minutes (4,15). After this period, specimens were rinsed with ultrapure water for 1 minute and dried with white tissue paper (16). Transparent adhesive strips (Durex Polisil TR 12X30, 3M, United States) were applied to the blocks so as to divide them in half, thus resulting in two parts of equal size (4 x 2 x 2 mm), with one of them representing the positive control group (which has not received brushing with any dentifrice) and the other comprising the experimental part.

•Brushing simulation

The test bodies were submitted to simulated brushing (MEV2, Odeme Biotechnology, Brazil), in which a load corresponding to 200 g was applied to the toothbrush (Oral-B Sensitive Ultra smooth- Ultra soft), according to the ISO14569 standard specification # - 1 (17). The dentifrice dilution was also performed according to the data of ISO 14569 specification # - 1, which recommends mixture of 2: 1 (m/v) of ultrapure water and dentifrice (60 mL of ultrapure water to 30 g of dentifrice) (17). In the experimental protocol, an eight-week period was simulated, with two daily brushings, being that the number and duration of brushings were done in order to reach the obliteration peak of the dentinal tubules, following the guidelines provided by dentifrice manufacturers. Each specimen was brushed according to 20 brushing cycles, comprised of two brushings a day for eight weeks, totaling 2,240 brushing cycles. This number was based on the estimate that each tooth is brushed for ten seconds in a two-minute brushing (16). The specimens were randomly divided into groups, as follows: G1 (n = 8) - brushing with dentifrice containing 8% arginine and calcium carbonate (Colgate Sensitive Pro-Relief®); G2 (n = 8), - brushing with dentifrice containing 8% strontium acetate (Sensodyne Rapid Relief®); G3 (n = 8), - brushing with the dentifrice containing 5% sodium calcium phosphosilicate (Sensodyne Repair & Protect®); G4 (n = 8), - brushing with the dentifrice base of calcium carbonate (Colgate Maximum Cavity Protection® - negative control). Upon completion of the brushing period, the adhesive tapes were removed with the aid of a medical clamp in order to analyze the surfaces of the samples. The specimens were washed with ultrapure water for one minute, and with the aid of ultrasonic tank for five minutes, in order to remove the remaining excess dentifrice, thus simulating mouth rinse, dried with white tissue paper and stored in Drying Oven Microprocessor (Q317M, Quimis, Brazil) (48.6º C for 12 hours).

•Dentin Analysis 

In order to be analyzed, the test bodies were taken to a low vacuum scanning electron microscope (MEV Phenom ProX/EDS - Phenom World, Eindhoven, Netherlands) in order to verify the presence and characteristics of dentinal tubules (16,17). Photomi-crographs were performed, providing magnification of 5,000 times in the control face and in the experimental face, and a magnification of 2,000 times in the control/experimental interface, as well as a three-dimensional image of the same region. By coupling the technique of Energy Dispersive Spectroscopy (EDS) to the scanning electron microscopy (SEM) analyses of the specimens were made to determine the chemical elements present in the dentinal tubules after application of desensitizing dentifrices, taking their formulations into consideration (18). Representative samples of each group were randomly selected, in which the dentin surface profiles were obtained with a contact profilometer (Talysurf i60, Taylor Hobson, United Kingdom), in order to check the potential of the abrasive dentifrice, by assessing the loss of surface dentin structure (17,19). Qualitative analysis of the photomicrographs and the topographic images of the dentin surface were performed.

-Statistical Analysis

The hydrodynamic size, polydispersity index and zeta potential of the particles present in the dentifrices were subjected to analysis of variance (ANOVA), followed by Tukey post hoc test, since all variables met the prerequisites regarding normality (Shapiro -Wilk, *p*> 0.05), homoscedasticity (Levene test for homogeneity of variances, *p*> 0.05) and independence (Durbin Watson test, *p* ~ 2.0). To compare the chemical elements present in each sample Student’s t-test for independent samples (*p* <0.05) was conducted. All statistical analyses were performed using the Statistical Package for Social Sciences software (SPSS® Inc., Chicago, United States), version 13.0, with a 95% significance level.

## Results

-Characterization of hydrodynamic size, polydispersity index and zeta potential of the particles of the dentifrices

Results regarding physical and chemical parameters of dentifrices were obtained by calculating the average of three readings and are shown in  Table 1.

**Table 1:**
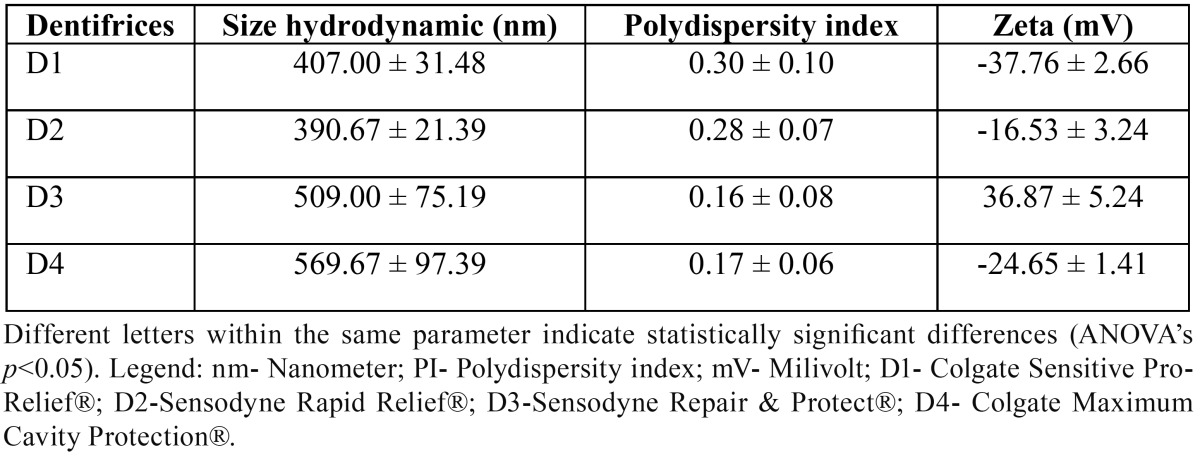
Results expressed as average ± standard deviation (n=3).

With regards to the hydrodynamic size, there was a statistically significant difference only between dentifrice D2 (Sensodyne Rapid Relief®) and dentifrice D4 (Colgate Maximum Cavity Protection®) in our study. Based on the average data presented in relation to the polydispersion index, values were statistically similar in the four dentifrices. All of them presented values of around 0.16 to 0.30, which indicates relative stability, since the optimal values would be smaller than 0.20. Zeta potential analysis shows better colloidal stability in solution for dentifrice D1 (Colgate Sensitive Pro-Relief®) and dentifrice D3 (Sensodyne Repair & Protect®) (*p* <0.05) when compared to D2 and D4. D2 and D4 are statistically similar with regards to this parameter.

-Evaluation of the presence and characteristics of the dentinal tubules by SEM

Regarding the presence and characteristics of dentinal tubules by SEM, three samples from each group were randomly selected so that a photomicrograph of the interface (control / experimental) could be performed, with a magnification of 2,000 times and 3D image. Photomicrographs of six samples of G1, G2, G3 and G4 were done with a magnification of 5,000 times, comprising a control face and an experimental face.

**Figure 2:**
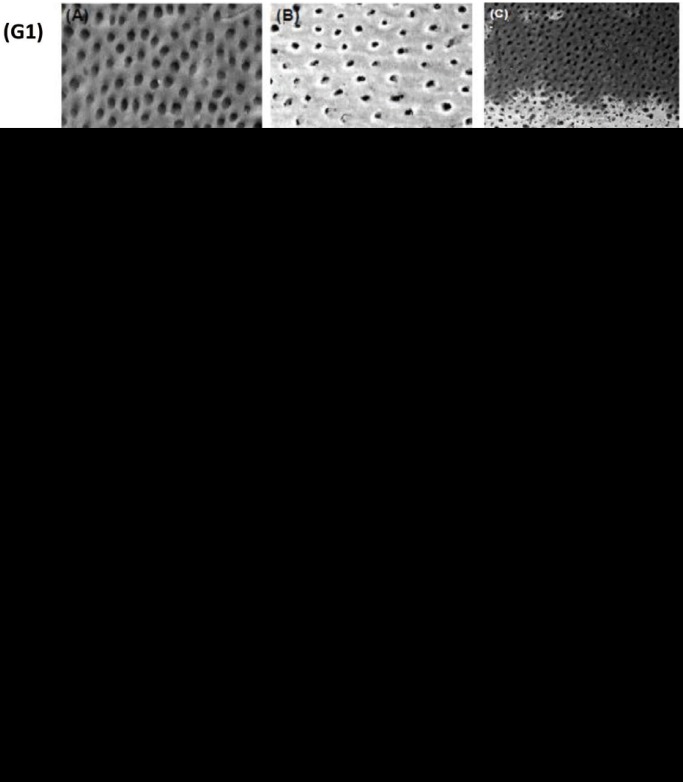
Photomicrographs of a representative specimen from each group. A) Control Face and B) Experimental face. Magnification of 5,000X. C) Interface (control / experimental interface), with magnification of 2,000X and (G1) brushing with Colgate Sensitive Pro-Relief®; (G2) brushing with Sensodyne Rapid Relief®; (G3) brushing with Sensodyne Repair & Protect®; and (G4) brushing with Colgate Maximum Cavity Protection®.

SEM representative images of the specimens from G1 (Fig. 2 - G1) show small obliteration of dentinal tubules in the experimental part (B) when compared to positive control (A). In the photomicrographs from G2 (Fig. 2- G2), there is clear deposition of oblite-rating particles in the dentinal tubules in the experimental area (B) when compared to the positive control surface (A). The images from G3 (Fig. 2 - G3), showed reduction in the diameter of the dentinal tubules in the experimental part (B) when compared to the positive control (A), as well as the formation of a layer on the dentin, thus occluding the dentinal tubules. Photomicrographs from G4 (Fig. 2- G4) are the negative control, since the dentifrice contains no active ingredient for DH. In that group, differences in the experimental regions (B) are minor when compared to the positive control surfaces (A). From the results obtained in our study, it is observed that with regards to the analysis by means of photomicrography, the desensitizing dentifrices succeeded in reducing the caliber of the dentinal tubules following, respectively, in a decreasing order of effectiveness, D3, D2 and D1. These results are solidified by evaluation through EDS.

-Evaluation of the chemical elements present in the dentin samples using EDS

**Figure 3:**
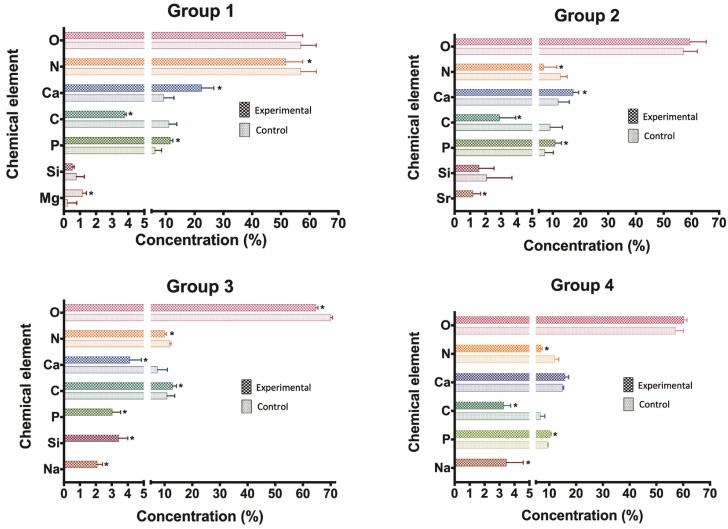
Chemical composition of the specimens: G1 (brushing with Colgate Sensitive Pro-Relief® dentifrice); G2 (brushing with Sensodyne Rapid Relief®); G3 (brushing with Sensodyne Repair & Protect® dentifrice); and G4 (negative control - brushing with Colgate Maximum Cavity Protection® dentifrice). * *p* <0.05 (experimental group compared to the control). Legend: O-oxygen; N-nitrogen; Ca-calcium; C-carbon; P-phosphorus; Si-silicon; Mg- magnesium; Sr- strontium; Na-sodium.

With the aid of the EDS technique, analyses of samples previously evaluated by SEM were performed in order to determine the chemical elements present in the dentinal tubules after the application of desensitizing dentifrices, taking into consideration their formulations and based on the positive control of each group. Figure 3 show, respectively, the average percentage of chemical elements present on the experimental and control surfaces in the specimens belonging to groups G1, G2, G3 and G4.

In the analysis of the chemical composition of Group 1 samples, a statistically significant difference (*p* <0.05) was verified between the control’s and experimental’s surfaces, mainly with respect to carbon, calcium, nitrogen, phosphorus and magnesium. Since carbon and calcium are the basic components of Colgate Sensitive Pro-Relief® dentifrice (8% arginine, calcium carbonate, 1.10% sodium monofluorophosphate; 1450 ppm fluoride), this confirms the discrete dentinal tubule obliteration seen in the photomicrographs of the experimental region in the same group. Evaluation regarding the chemical elements present in the specimen from Group 2 shows a statistically significant difference (*p* <0.05) in the experimental faces when comparing to the positive control faces for carbon, calcium, nitrogen, phosphorus and strontium. These data are corroborated taking into account the formulation of Sensodyne Rapid Relief® dentifrice (8% strontium acetate, calcium carbonate, sodium fluoride; 1,040 ppm fluoride), and the clear deposition of obliterating particles in the dentinal tubules of the experimental regions. By checking the chemical elements of specimens from Group 3, a statistically significant difference (*p*<0.05) between the positive and the experimental control regions for all chemical elements present in oxygen; carbon; calcium; nitrogen; phosphorus; silicon and sodium, samples was noticed, which is solidified by the dentifrice chemical composition (5% calcium phosphosilicate and sodium and 1426 ppm fluoride) and by the reduction in the diameter of dentinal tubules in the experimental part when compared to the control, besides the formation of a layer over the dentin, as verified in the SEM images from Group 3. Analysis of the chemical elements of specimens from Group 4 (brushing with Colgate Maximum Cavity Protection® - negative control) showed a statistically significant difference (*p* <0.05) in the experimental faces when compared to the positive controls as for oxygen, nitrogen, carbon, phosphorus and sodium, whose results are based on the dentifrice formulation (calcium carbonate and 1,450 ppm fluoride) and slight differences were noticed in the experimental areas when compared to the surfaces of the positive control in the SEM images from Group 4.

-Analysis of the loss of dentin surface structure employing profilometry

**Figure 4:**
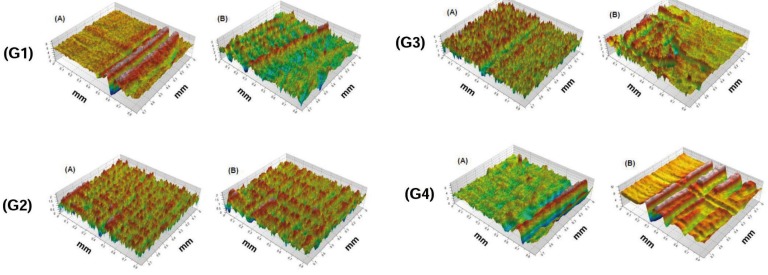
3D topographic image of the interface (control / experimental interface) of two specimens from G1 (brushing with Colgate Sensitive Pro-Relief®); G2 (brushing with Sensodyne Rapid Relief®); G3 (brushing with Sensodyne Repair & Protect®); and G4 (negative control - brushing with Colgate Maximum Cavity Protection®). (A) Specimen 1; and (B) Specimen 2.

Two representative samples from each group were used for profilometry analysis, in order to assess the loss in the structure of the dentinary surface. Figure 4 shows: G1 (the presence of peaks and valleys in the experimental region with great variation in height in relation to the positive control face, G2 slight variation between the experimental area and the positive control face; G3 extreme symmetry, in which the experimental region ranged from 2-0 mm, while the positive control remained leveled at 1.5mm in both (a) as in (B) and G4, presence of peaks and valleys showing greater height difference in the experimental area when compared to the positive control surface.

Therefore, a higher degree of surface wear was verified in the region treated with the following dentifrices, in this respective order: D4; D1; D2; e D3, being that in the last two dentifrices, certain superficial regularity was observed between the control and the experimental regions.

## Discussion

The hydrodynamic size shows the size of dentifrice particles present in the solution (20). The smaller the dentifrice particles, the easier is their penetration into the dentinal tubules. In this sense, although the results showed close particle diameters among the groups, D2 exhibited the lower values, indicating that this dentifrice could have better absorption by the dental tissue. Also with regards to average particle size, the polydispersion index (PI) indicates the size distribution of the dentifrice particles and generally values lower than 0.20 for colloidal suspensions are considered to be good stability indicators (20), and within this context D3 and D4 meet this criteria. The PI presented by D1 and D2 are higher than 0.20, but lower than 0.30, indicating that they also possess a good, but not ideal, distribution of size of their particles. Zeta potential was another parameter evaluated in our study. This parameter reflects the surface charge of the dentifrice particles and can be influenced by the composition of the particle, the dispersing medium, the pH and the ionic strength present in the solution (20). Generally, particles having zeta potential values of at least ± 25 mV have good colloidal stability when in solution (20), which was observed in D1, D3 and D4. Further, the higher the stability, the lower the reactivity of the particles, and in the case of desensitizing dentifrices, particles should be more reactive, since dentifrice oligoelements must be released from their matrixes and deposited into the tubules – in this case, D2 would present a better effect than the others, as its surface charge was lower. That is, a high zeta potential can hinder the transfer of oligoelements from the dentifrice to the tooth. It should be noted that these results concern the hydrodynamic potential of the products, due to the fact that the readings are made with the same as solution / suspension. However, daily usage and storage of the products are done under anhydrous conditions. In this context, a future perspective for assessing short and long-term stability is emphasized, in order to check whether zeta potential results have a major influence on dentifrice shelf life. With respect to the daily use by population, the most commonly used dentifrices are those ones based on 8% strontium acetate (D2), 8% arginine and calcium carbonate (D1) and 5% calcium phosphosilicate and sodium (D3). Strontium salts precipitate insoluble metal compounds on the tooth surface, fully or partially blocking open dentinal tubules. Alternative explanations described in the literature point to effects regarding nerve depolarization or replacement of the calcium present in the hydroxyapatite structure by strontium, due to their similar chemical structure, in order to strengthen demineralized dentine (5,6,10). Studies with desensitizing dentifrices have often shown contradictory results, mostly due to differing methodologies (8). On the grounds of these contradictions observed, we opted to follow the ISO standard protocols, in order to have more significant data. However, in the following paragraphs we attempted to compare our findings with the ones from other authors, regardless of the methodology used. According to Cummis (21), there is no conclusive evidence to corroborate the fact that dentifrices containing strontium salts have sufficient effectiveness to promote immediate relief from DH. Furthermore, clinical evidence related to long-term relief during the routine use of the dentifrice (twice a day) when brushing is doubtful. Now dentifrices with the combination of calcium carbonate and arginine attempt to emulate the DH reduction biological process which occurs in the presence of calcium, phosphate and salivary glycoproteins. Arginine, a naturally occurring amino acid in saliva, acts simultaneously with calcium carbonate and phosphate in order to create an occlusive adhesive (plug-in) in the dentinal tubules, thus preventing fluid flow (4-6,11,21). Cummis (21) states that dentifrice containing arginine and calcium carbonate has been clinically proven to provide immediate relief after a single direct topical application. In their study, the aforementioned dentifrice promoted a significantly higher percentage of reduction in the fluid flow after application, when correlated with dentifrice containing strontium acetate, and the effects were maintained after an acid challenge. Now Patel (22), in a hydraulic conductance study, showed that the dentifrice containing arginine and calcium carbonate is more effective in the occlusion of open dentinal tubules and in the reduction of dentinal fluid flow when compared to the dentifrice containing strontium acetate, wherein the occlusion achieved with the use of the former is resistant to an acid challenge. Schiff (23) found a statistically significant improvement in the average scores of both tactile and air blast hypersensitivity, corresponding to 51.3% and 39.4%, respectively, due to the use of dentifrice with arginine and calcium carbonate and with strontium acetate. In the second stage of the study, individuals who had brushed their teeth with dentifrice containing arginine and calcium carbonate during the first eight weeks, after that period started to brush with another dentifrice, showing no improvements in the average data regarding tactile hypersensitivity or hypersensitivity to air blast. The opposite was also tested, resulting in improvements (35.3% to 40.3%) though. In disagreement with those results and confirming the findings of this study, Davies (10), in a study conducted with dentin samples, concluded that the dentifrice containing strontium acetate performs a better function of obliteration of dentinal tubules than the one containing arginine and calcium carbonate. The author also stated that the initial strontium-based dentifrice formulations have not always shown statistical significance in the reduction of DH pain, however, greater efficacy has been shown for recently developed strontium-based dentifrices. Regarding D3, reports in the literature are similar to the results found in our study. Bioglasses consist of particular proportions of silicon dioxide (SiO2), sodium oxide (Na2O) and phosphorus pentoxide (P2O5) (6). The action mechanism for the DH reduction is the occlusion of open dentinal tubules, by promoting the growth of new calcium phosphate crystals on the surface of the tooth (5,6,15) and precipitate the hydroxycarbonate apatite layer (5,15). In research conducted by Joshi, Gowda and Joshi (24), samples treated with dentifrice containing NovaMin® technology (D3), showed a layer from 2 to 3 mm thick with large crystalline particles of tubule occlusion in some regions. The same authors stated that this showed to be the most effective desensitizing dentifrice. Satyapal* et al.* (25), through an *in vivo* study, found a statistically significant reduction of DH to air and water stimuli, at three and six weeks of D3 usage. In an *in vivo* study, West * et al.* (26) demonstrated the capacity D3 has to occlude the dentinal tubules and these remain occluded even after an acid challenge. Parkinson and Wilson (27) showed that D3 achieved excellent results, not only by forming a layer on the dentin, thus occluding the dentinal tubules, but also by providing a tougher surface, proven through the microhardness test. Through a study using the SEM, Earl * et al.* (28), confirmed the existence of a layer formed on the dentin when calcium sodium phosphosilicate (D3) reacted with the artificial saliva. Within this dialectic, Olley et al. (3) and Seong * et al.* (29) concluded that both D2, and D1 significantly reduce the dentinal tubule caliber, but the dentifrice with arginine and calcium carbonate is more susceptible to acid changes. Therefore, the occlusion pro-vided by D2 is greater in the presence of an acidic diet. By means of EDS analysis, Davies * et al.* (10), revealed that the concentrations of calcium, phosphorous and oxygen increased in the dentin samples treated with D1. In specimens brushed with D2, the presence of calcium, phosphorus and sulfur were highlighted. These results corroborate the findings in our study with respect to the increase in the average of the aforementioned chemicals, but this increase was not statistically significant. However, the results are in disagreement in terms of sulfur appearance when using D2 and in terms of the percentage of oxygen growth when brushing with D1. To sum up, joint data analysis shows that the desensitizing dentifrices demonstrated superior results with regards to the obliteration of dentinal tubules, with respect to positive and negative controls, indicating that they could have *in vivo* efficacy which must be proven in further studies.
